# Pomegranate Extract Modulates Oxidative Stress by Reducing Basal ROS Levels and Protecting White Blood Cells from Induced Oxidative Damage in Aging Mice

**DOI:** 10.3390/ijms26135957

**Published:** 2025-06-20

**Authors:** David Verdú, Alicia Valls, Marta Serna-García, Guadalupe Herrera, Mustafa Ezzeddin-Ayoub, Maria D. Mauricio, José Viña, Eva Serna

**Affiliations:** 1 Department of Physiology, Faculty of Medicine and Dentistry, CIBERFES, INCLIVA Biomedical Research Institute, University of Valencia, 46010 Valencia, Spain; david.verdu@uv.es (D.V.); alicia.valls@uv.es (A.V.); m.dolores.mauricio@uv.es (M.D.M.); jose.vina@uv.es (J.V.); 2 MODULAhR Group, University of Valencia, 46010 Valencia, Spain; marta.serna@universidadeuropea.es; 3 Department of Dentistry, Faculty of Health Sciences, Universidad Europea de Valencia, 46010 Valencia, Spain; 4 Flow Cytometry Unit, UCIM-IIS INCLIVA, Biomedical Research Institute, University of Valencia, 46010 Valencia, Spain; guadalupe.herrera@uv.es; 5 Micro PET-CT Unit, UCIM-IIS INCLIVA, Biomedical Research Institute, University of Valencia, 46010 Valencia, Spain; mustafa.ezz@uv.es

**Keywords:** oxidative stress, aging, pomegranate extract, flow cytometry

## Abstract

Aging is associated with increased oxidative stress, which contributes to cellular dysfunction and age-related diseases. Pomegranate extract (PE), rich in antioxidant polyphenols, may help mitigate oxidative damage. This study evaluated whether PE supplementation modulates oxidative stress by reducing reactive oxygen species (ROS) levels in white blood cells of aging mice. Aged mice (18 months) were supplemented with PE for four months, and cytoplasmic and mitochondrial ROS levels were assessed in leukocytes under basal conditions and oxidative stress conditions induced by tert-butyl hydroperoxide (t-BHP) using flow cytometry. Our results indicate that aged mice exhibit increased basal ROS levels in both the mitochondrial and cytoplasmic compartments, which were mitigated by PE supplementation. Furthermore, PE reversed the increase in hydrogen peroxide levels induced by τ-BHP and protected neutrophils by reducing mitochondrial ROS levels. These findings suggest that PE supplementation modulates the oxidative stress response, potentially improving immune function in aging. Given the central role of oxidative stress in age-related decline, PE may represent a valuable nutritional strategy to promote healthy aging.

## 1. Introduction

Oxidative stress is a physiological condition characterized by an imbalance between the production of reactive oxygen species (ROS) and the body’s ability to neutralize them. ROS are oxygen-derived by-products that include hydroxyl radical, superoxide radical, and hydrogen peroxide. Oxidative stress has been implicated in various chronic diseases, including cardiovascular disorders, neurodegenerative conditions, and the aging process [1,2,3,4].

Aging is a complex biological phenomenon associated with the gradual decline of cellular function and increased susceptibility to various age-related diseases [5,6]. One of the contributing factors to the aging process is the cumulative impact of oxidative stress on cells and tissues over time. As cells age, they become more vulnerable to oxidative damage, leading to cellular dysfunction and, ultimately, the manifestation of age-related conditions [7].

Among the experimental tools commonly used to study oxidative damage, tert-butyl hydroperoxide (t-BHP) is frequently employed to mimic oxidative stress conditions in vitro and in vivo. t-BHP induces the formation of ROS and lipid peroxidation, providing a useful model to evaluate the antioxidant capacity of compounds under controlled oxidative challenges [8,9,10]. In parallel, malondialdehyde (MDA) is a well-established biomarker of lipid peroxidation and oxidative stress, widely used in aging studies to quantify membrane damage induced by ROS [10,11].

The free radical theory of aging provided a background for many research groups working in this field [12]. Furthermore, mitochondria play a main role in the aging process, as proposed by the mitochondrial theory of aging that links mitochondrial dysfunction to aging [13]. Over time, the mitochondrial DNA can accumulate mutations. Unlike nuclear DNA, mitochondria have their own DNA, and this DNA is more susceptible to damage due to its proximity to ROS generated during energy production. The accumulation of mitochondrial DNA mutations can lead to impaired mitochondrial function, and aging mitochondria become less efficient at generating ATP through oxidative phosphorylation. This inefficiency leads to increased ROS production, further damaging cellular components and contribute to the aging process [14]. Mitochondrial dysfunction has been linked to age-related diseases such as neurodegenerative disorders, cardiovascular diseases, and metabolic disorders [15,16].

Pomegranate (*Punica granatum*) has gained significant attention in recent years due to its potential health benefits, particularly its antioxidant properties [17,18,19,20]. Pomegranate is rich in bioactive compounds such as polyphenols, flavonoids, and anthocyanins, which contribute to its potent antioxidant activity. Antioxidants play an important role in combating oxidative stress, and studies suggest that pomegranate extract may not only help mitigate oxidative stress associated with aging but also exhibit anti-aging properties by promoting cellular health and resilience [21]. Interventions to support physiological function in aging include regular physical exercise, caloric restriction, and prebiotics and probiotics supplementation [22,23,24,25]. In this context, exploring the relationship between pomegranate extract supplementation and oxidative stress opens opportunities for understanding its impact on cellular health and its potential role in preventing or ameliorating age-related disorders.

The aim of this study was to assess the potential of pomegranate extract (PE) supplementation in reducing basal ROS production and protecting against inducible oxidative stress in white blood cells (WBC) of aging mice.

## 2. Results

### 2.1. Effect of Pomegranate Extract on White Blood Cell Count and Lipid Peroxidation in Plasma

**Figure 1:**
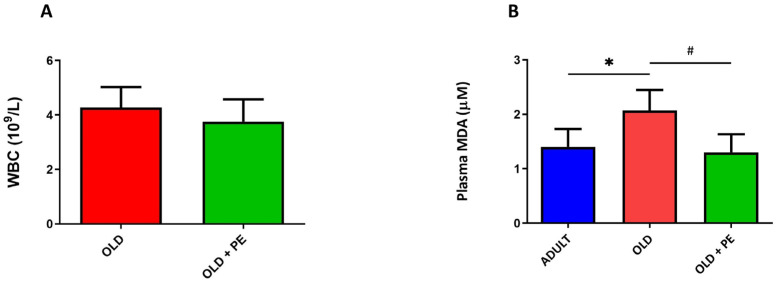
Effect of PE on total WBC in blood (**A**) and lipid peroxidation (MDA) in plasma (**B**). Results are expressed as mean ± SEM (*n* = 4–6 per group). * *p* < 0.05 vs. Adult group # *p* < 0.05 vs. Old group.

No differences were found in the WBC count when comparing the 22-month-old non-supplemented and supplemented groups. However, aged mice exhibited higher MDA levels, and this increase was reversed in the group supplemented with PE (Figure 1).

### 2.2. Study of Intracellular Basal ROS Levels with PE Supplementation

**Figure 2:**
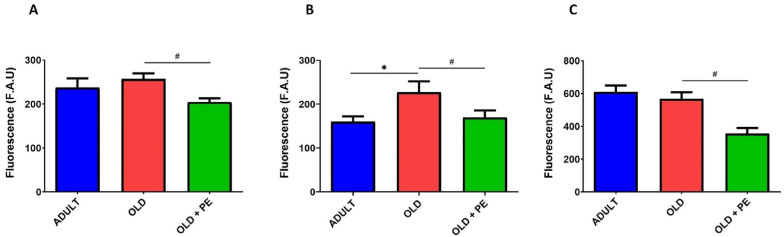
Levels of intracellular ROS in WBC measured using the H_2_DCF-DA fluorochrome. Quantification of intracellular ROS levels in total leukocytes (**A**), lymphocytes and monocytes (**B**), and neutrophils (**C**) from 10-month-old (Adult group) and 22-month-old mice (Old group) with and without PE supplementation. Data are expressed as mean ± SEM (*n* = 4–7 per group). * *p* < 0.05 vs. Adult group # *p* < 0.05 vs. Old group.

**Figure 3:**
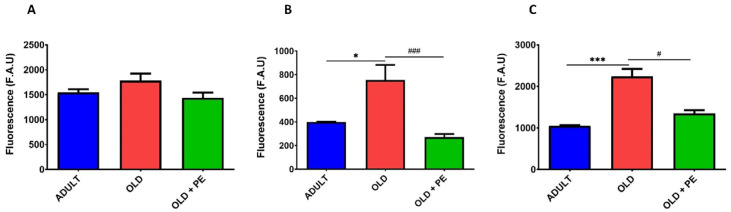
Levels of hydrogen peroxide in WBC measured using the DHR123 fluorochrome. Quantification of hydrogen peroxide in total leukocytes (**A**), lymphocytes and monocytes (**B**), and neutrophils (**C**) from 10-month-old (Adult group) and 22-month-old mice (Old group) with and without PE supplementation. Data are expressed as mean ± SEM (*n* = 4–7 per group). * *p* < 0.05 vs. Adult group; *** *p* < 0.001 vs. Adult group; # *p* < 0.05 vs. Old group; ### *p* < 0.001 vs. Old group.

**Figure 4:**
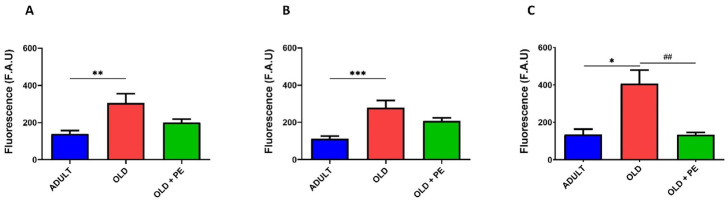
Mitochondrial levels of hydrogen peroxide in WBC measured using the MitoPY1 fluorochrome. Quantification of hydrogen peroxide in total leukocytes (**A**), lymphocytes and monocytes (**B**), and neutrophils (**C**) from 10-month-old (Adult group) and 22-month-old mice (Old group) with and without PE supplementation. Data are expressed as mean ± SEM (*n* = 4–7 per group). * *p* < 0.05 vs. Adult group; ** *p* < 0.01 vs. Adult group; *** *p* < 0.001 vs. Adult group; ## *p* < 0.01 vs. Old group.

We analyzed intracellular ROS using dichlorodihydrofluorescein diacetate (H_2_DCF-DA) (Figure 2), dihydrorhodamine 123 (DHR123) (Figure 3), as well as mitochondrial ROS using mitochondria peroxy yellow 1 (MitoPY1) (Figure 4) as fluorochromes in WBC from adult (10 months), aged mice (22 months), and aged mice supplemented with PE. Lymphocytes and monocytes were analyzed together, assuming a single area on the Side Scatter (SSC) and Forward Scatter (FSC) plots while neutrophils were differentiated on their distinct morphology and complexity.

In Figure 2, we show that the fluorescence was reduced in supplemented aged mice (Figure 2A). This reduction was also observed in monocytes and lymphocytes populations (Figure 2B) and as well as in neutrophils (Figure 2C). These findings suggest that PE plays a protective role by preventing the increase in intracellular ROS in WBC in aged mice.

Specifically, hydrogen peroxide levels were detected by DHR123 fluorochrome (Figure 3). ROS levels were significantly higher in lymphocytes and monocytes (Figure 3B), and neutrophils (Figure 3C) from the non-supplemented aged mice compared to the adult group (Figure 3). However, PE supplementation in aged mice reversed these increases, bringing ROS levels back to levels similar to those observed in the adult group.

Using a fluorochrome specific for mitochondrial hydrogen peroxide, increased levels were observed in total leukocytes as such as lymphocytes and monocytes, and neutrophils of aged mice (Figure 4). However, PE supplementation significantly reduced mitochondrial ROS, especially in neutrophils (Figure 4C) but not in lymphocytes and monocytes (Figure 4B).

Therefore, these findings confirm that PE supplementation mitigates the age-associated increase in both cytoplasmic and mitochondrial ROS production and may contribute to the modulation of both the adaptive and innate immune responses.

### 2.3. Study of Intracellular ROS Levels Induced with t-BHP in Aged Mice with PE Supplementation

We evaluated both cytoplasmic and mitochondrial basal ROS levels in total leukocytes, neutrophils and lymphocytes, and monocytes from aged mice (22 months, Old group) compared with aged mice supplemented with PE. To further assess oxidative stress response, blood samples from both aged groups were treated with the oxidant agent t-BHP and compared to non-oxidized samples.

**Figure 5:**
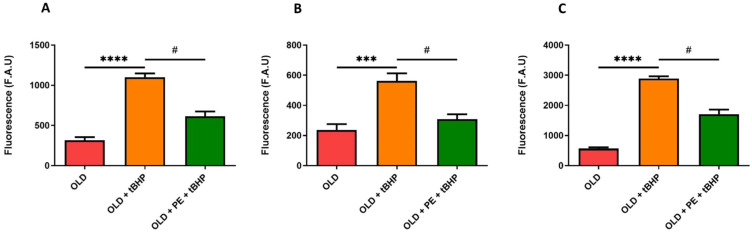
Levels of intracellular ROS in WBC of aged mice induced with t-BHP using the H_2_DCF-DA fluorochrome. Quantification of intracellular ROS levels induced with 50 µM of t-BHP in total leukocytes (**A**), lymphocytes and monocytes (**B**), and neutrophils (**C**) from 22-month-old mice (Old group) with and without PE supplementation. Data are expressed as mean ± SEM (*n* = 4–7 per group). *** *p* < 0.001 vs. Adult group; **** *p* < 0.0001 vs. Adult group; # *p* < 0.05 vs. Old group.

Upon oxidative damage induction with t-BHP, PE supplementation resulted in a reduction in total intracellular ROS levels, as measured by fluorescence using H_2_DCF-DA fluorochrome (Figure 5A). Specifically, the increase in ROS levels induced by t-BHP was reversed in lymphocytes and monocytes (Figure 5B), as well as in neutrophils (Figure 5C), in PE-supplemented mice.

**Figure 6:**
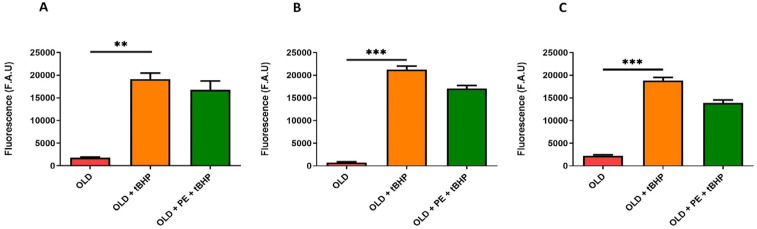
Levels of hydrogen peroxide in WBC of aged mice induced with t-BHP using the DHR123 fluorochrome. Quantification of hydrogen peroxide induced with 50 µM of t-BHP in total leukocytes (**A**), lymphocytes and monocytes (**B**), and neutrophils (**C**) from 22-month-old mice (Old group) with and without PE supplementation. Data are expressed as mean ± SEM (*n* = 4–7 per group). ** *p* < 0.01 vs. Adult group; *** *p* < 0.001 vs. Adult group.

Unexpectedly, this reduction in ROS with the DHR123 fluorochrome in supplemented group was not observed with t-BHP induction (Figure 6).

**Figure 7:**
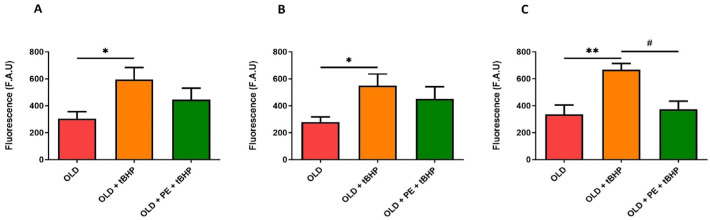
Mitochondrial levels of hydrogen peroxide in WBC of aged mice induced with t-BHP using the MitoPY1 fluorochrome. Quantification of mitochondrial levels of hydrogen peroxide induced with 50 µM of t-BHP in total leukocytes (**A**), lymphocytes and monocytes (**B**), and neutrophils (**C**) from 22-month-old mice (Old group) with and without PE supplementation. Data are expressed as mean ± SEM (*n* = 4–7 per group). * *p* < 0.05 vs. Adult group; ** *p* < 0.01 vs. Adult group; # *p* < 0.05 vs. Old group.

In addition, mitochondrial ROS, detected with MitoPY1 fluorochrome, increased as expected with t-BHP treatment in all leucocytes (Figure 7). A remarkable result is that, in neutrophils (Figure 7C), PE supplementation exerts a protective effect against t-BHP oxidation, maintaining the fluorescence intensity similar to that of 22-month-old mice and lower than in the oxidative damage group. However, PE supplementation was not able to reduce mitochondrial hydrogen peroxide levels in leukocytes, lymphocytes, and monocytes (Figure 7A,B).

Based on these findings, it can be concluded that PE is effective in reducing ROS levels in aged mice subjected to induced oxidative damage. Additionally, in the t-BHP group, PE reduces mitochondrial hydrogen peroxide levels in neutrophils, whereas no reduction is observed in the non-supplemented group.

## 3. Discussion

A diet high in antioxidants contributes to successful aging by protecting against oxidative damage, reducing the risk of chronic diseases, improving the ability to maintain physical, cognitive and social health, while adapting to age-related changes to preserve quality of life and well-being [26]. Gerontologists agree that with age, takes place an age-related oxidative stress generated by a combination of increased production of free radicals, decreased antioxidant levels, diminished activities of antioxidant enzymes, and impaired repairs of oxidative damage [27]. Moreover, leukocyte capacities are strongly influenced by the antioxidant/oxidant balance, because immune cells produce ROS to support their functions. From this point of view, the antioxidant levels of these cells are very important to maintain redox homeostasis and, therefore, an adequate function, especially during oxidative stress situations such as aging. Thus, adequate amounts of neutralizing antioxidants are required to prevent damage to the immune cells themselves [28].

Our findings suggest that PE supplementation in aged mice reduces oxidative stress, as evidenced by lower basal ROS levels and protection against induced oxidative damage in WBC. Aged mice exhibited increased basal levels of intracellular and mitochondrial ROS in blood cells, consistent with previous evidence linking oxidative stress to aging-related cellular dysfunction [29,30].

Firstly, levels of intracellular peroxides, detected through fluorescence using H_2_DCF-DA fluorochrome, were reduced in leukocytes, monocytes and lymphocytes, and neutrophils from PE supplemented group compared to the non-supplemented mice group in basal conditions and with t-BHP. t-BHP can induce lipid peroxidation, mitochondrial dysfunction, and cellular damage, simulating oxidative stress conditions [31].

During aging, the antimicrobial capacity of neutrophils decreases due to shorter life span, reduced chemotaxis, phagocytosis, and the capacity to generate ROS [32,33,34]. In mice, aging-related defects in intracellular bacterial killing have been linked to a decline in neutrophil ROS production [35,36].

It should also be noted that H_2_DCF-DA is a general marker for ROS and does not differentiate between specific species (e.g., hydrogen peroxide, hydroxyl radical). This may limit its precision when studying the specific effects of different ROS on aging, as antioxidant enzyme activity in aged or damaged cells may vary, potentially influencing the interpretation of results with H_2_DCF-DA due to additional factors that can affect fluorescence.

Therefore, other specific ROS markers were selected, DHR123, as a valuable tool for quantifying oxidative stress in living cells [37]. Accumulated damage is a hallmark of aging, as cells lose their repair capacity over time. In our study, 22-month-old mice exhibited higher concentrations of H_2_O_2_ and O_2_^−^ in their WBC, which oxidized DHR123 to rhodamine producing a greater signal compared with 10-month-old mice. Specifically, an increase in ROS in neutrophils measured using this fluorochrome has been associated with aging phenotypes and a pro-inflammatory gene expression pattern which contributes to a shorter lifespan. Notably, aged mice supplemented with PE showed lower ROS concentrations, reflected by a reduced DHR123 fluorescence intensity. However, this effect could not be reversed when t-BHP was used to induce greater oxidative damage in the leukocyte population. In the same way as our findings, previous studies have demonstrated that PE can decrease ROS levels in cultured cells exposed to oxidative stress, with DHR123 being used in various assays to confirm its antioxidant efficacy [38,39,40].

Additionally, similar antioxidant effects have been reported for compounds such as N-acetylcysteine, resveratrol, vitamins C and E, curcumin, melatonin, and quercetin. These agents have shown the ability to reduce ROS production in leukocytes from aged mice and improve immune function [9,41,42,43]. For example, resveratrol and vitamin E have been reported to decrease oxidative damage and improve mitochondrial function in immune cells [44]. The similarity of our results with those obtained with other antioxidant strategies reinforces the relevance of PE as a dietary intervention for redox regulation during aging.

Mitochondrial ROS play a critical role in the aging process, inflammatory responses, and metabolic disorders [45,46,47]. By using MitoPY1, a fluorescent probe specifically designed to detect hydrogen peroxide generated in mitochondria, we found that PE is capable of modulating the innate immune response, as it reduced the basal levels of neutrophils that are elevated with aging, as well as those induced by oxidative damage with t-BHP.

In line with oxidative stress, another relevant hallmark of aging is cellular senescence. Cellular senescence is a state of permanent cell cycle arrest associated with morphological and metabolic alterations, as well as with the acquisition of a pro-inflammatory phenotype known as SASP (senescence-associated secretory phenotype) that contributes to tissue dysfunction and inflammaging [8,48,49]. Oxidative stress is one of the major inducers of senescence, and antioxidant interventions such as PE could potentially modulate this process. However, we did not assess senescence markers in this study due to technical limitations (fresh blood requirement for SA-β-gal analysis). Nevertheless, we consider this an important avenue for future investigation.

To conclude, supplementation with PE significantly reduced ROS levels WBC both under basal conditions and following exposure to an oxidative challenge (t-BHP). Notably, mitochondrial H_2_O_2_ levels in neutrophils were also reduced in PE-supplemented mice, further supporting its role in oxidative stress protection. These protective effects are likely attributable to PE’s high content of polyphenols, flavonoids, and anthocyanins—compounds with well-documented antioxidant properties [17,18]. These bioactive molecules may enhance endogenous antioxidant defenses and reduce ROS accumulation, thereby contributing to cellular homeostasis in aging organisms and promoting resilience against oxidative stress.

Although our study focused on ROS levels as indicators of oxidative status, future studies should address the involvement of specific antioxidant defense mechanisms, such as the activity or expression of key enzymes like superoxide dismutase, catalase, or glutathione peroxidase, to further elucidate the molecular pathways underlying PE’s protective effects. Moreover, considering the tight interplay between oxidative stress and inflammation in aging, it would be of great interest to investigate whether PE supplementation also exerts anti-inflammatory effects.

Environmental factors, such as pollution, further exacerbate oxidative stress and inflammation, both of which are closely associated with aging and age-related diseases. The protective effects of PE may extend beyond natural aging, offering potential benefits in mitigating oxidative damage caused by environmental pollutants and enhancing its relevance as a nutritional strategy. Given the importance of reducing oxidative stress in immune cells, PE supplementation may help preserve immune function and contribute to overall health in aged individuals, especially in the context of aging-related inflammation. PE may also play a key role in helping organisms better adapt to physiological and environmental stressors associated with aging.

Future studies should explore whether PE supplementation improves immune function in other tissues or enhances resistance to infections and age-related inflammatory diseases. These findings support the potential of PE as a nutritional strategy to promote healthy aging, although further research is needed to elucidate its mechanisms of action and long-term benefits.

Pomegranate stands out as one of the fruits with the highest antioxidant capacity, primarily due to its richness in polyphenols such as punicalagin and ellagic acid [50,51,52]. While various compounds and functional foods have been proposed to reduce oxidative stress, the use of PE presents unique advantages, including its high bioavailability and broad biological activity. Importantly, recent studies have suggested beneficial effects of pomegranate not only in oxidative stress modulation but also in cognitive and physical function, which are key aspects of healthy aging [53,54,55,56].

What distinguishes our study from previous research is the dual approach evaluating both basal intracellular ROS levels and induced oxidative damage in WBC of aged mice. This approach has been rarely addressed in prior studies, and our results provide novel insights into how PE supplementation contributes to redox homeostasis at the cellular level during aging. Furthermore, we highlight the potential role of PE not only in preserving immune function in aging but also as a sustainable and accessible dietary strategy to enhance resilience against physiological and environmental stressors.

Nevertheless, a limitation of our study is that only male mice were used, which may restrict the generalizability of the findings, given that sex-based differences in oxidative stress and immune responses have been described. Future studies including both sexes will be necessary to fully understand the effects of PE across the aging population.

## 4. Materials and Methods

### 4.1. Experimental Animals

The animal study was approved by the Ethics Committee for Research and Animal Welfare of the University of Valencia (License reference: A20201110122413) and authorized by the Regional Government of Valencia (Authorization number: 2021/VSC/PEA/0006). Animals were housed at the Animal House Core Facility, Central Research Unit (UCIM-IIS) Faculty of Medicine and Dentistry, University of Valencia, under standard conditions (12 h light/dark cycle, 22 ± 2 °C, ad libitum access to food and drink). For the duration of the study, the mice were fed a standard diet, specifically the ENVIGO^®^ (Barcelona, Spain) Teklad Global 14% Protein Rodent Maintenance Diet (Sterilizable), which ensures consistency and reproducibility in the study.

Seventeen male C57BL6 (wild type, WT) mice were used in this study. Eighteen-month mice were assigned to one of two groups: control group or PE supplementation (150 mg/kg/day) group. The PE dosage was adjusted every three days based on liquid intake and body weight, and treatment lasted for 16 weeks. At the age of 22 months, animals were killed, and blood samples were collected for further analysis. Samples of WT mice (10 months) were used as an aging process control defined as an adult group.

### 4.2. Pomegranate Extract Supplementation

The natural concentrated extract of whole pomegranate used in this work is Pomanox^®^ P30, obtained through a water-based extraction process, was provided by Euromed (Barcelona, Spain). Pomanox^®^ P30 was prepared according to the European Patent EP1967079 [57] with a technology that utilizes only aqueous solutions avoiding the use of organic solvents. For manufacturing Pomanox^®^ P30, freshly harvested pomegranate fruits growing in the Spanish Mediterranean area are used. The steps of manufacturing are an extraction of water soluble compounds including punicalagins in aqueous solution, a separation of aqueous solution from pomegranate paste, a step of adsorption chromatography, a concentration by nanofiltration and finally pomegranate aqueous extract is brought to a solid form by spray drying. A detailed composition and analytical characterization of Pomanox^®^ P30 is provided in the Supplementary Materials (Table S1).

The extract is completely water-soluble and was administered via drinking water. The dosage of 150 mg/kg/day was selected based on human equivalent dose (HED) calculations using body surface area normalization, following the standard method described by [58]. This corresponds to an approximate human dose of 750 mg/day, aligning with effective doses used in previous clinical studies with Pomanox^®^ in humans [59].

### 4.3. Blood Collection and Sample Processing

**Figure 8:**
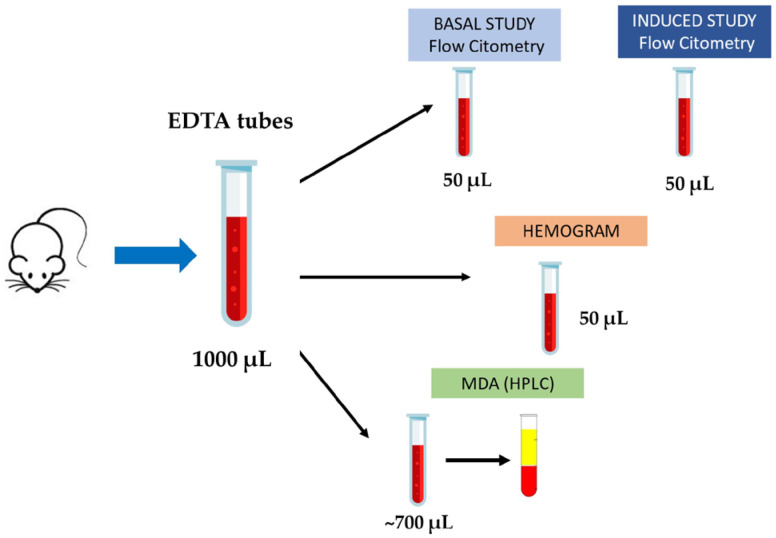
Workflow of blood collection and sample processing.

After deep anesthesia with isoflurane, mice were killed by exsanguination through the inferior vena cava, and approximately 1000 µL of blood was collected into EDTA tubes. Four aliquots were prepared: 100 µL were used for flow cytometry studies (50 µL for the baseline study and 50 µL for the induction study), 50 µL were used for hematological analysis (hemogram), and the remaining volume was processed to obtain plasma for subsequent MDA quantification by high-performance liquid chromatography (HPLC) (Figure 8).

All procedures were performed simultaneously, with one researcher assigned to each experimental protocol, and blood samples were processed immediately after collection to ensure sample integrity.

### 4.4. Hematological Analysis and Lipid Peroxidation Determination

Hematological parameters were obtained using the veterinary hematology analyzer HT-5 (Scil Animal Care Company, Heska group, Viernheim, Germany) located at the Central Unit for Research in Medicine (UCIM-IIS), Faculty of Medicine, University of Valencia. For each measurement, 20 µL of blood were mixed with 4 µL of EDTA and gently homogenized before analysis. The device provides a hematology count in less than one minute.

Lipid peroxidation determination as MDA levels was determined in plasma samples using HPLC in mice aged 10 months (Adult) and 22 months of age (Old) with or without supplementation, as described previously [60].

### 4.5. Flow Cytometry Analysis

**Figure 9:**
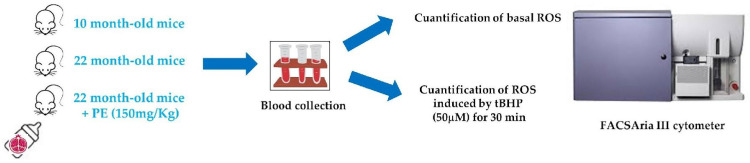
Summary of the experimental model used for flow cytometry analysis with PE supplementation.

The experimental model used is described in Figure 9.

All flow cytometry assays were performed using the FACSAria III cytometer (BD Biosciences, San Jose, CA, USA). The software for data collection was FACSDiva 4.0 while the offline analysis of the data was conducted with FLOWJO V.10.1 software. The events acquired per sample were 10,000 as a stopping condition, with a slow flow rate to avoid the appearance of doublets.

The fluorochromes used for the study of the oxidant species were as follows:

1. Dichlorodihydrofluorescein diacetate (H_2_DCF-DA) (5 mM, fluorescent 2,7-dichlorofluorescein DCF, λ excitation = 488 nm, λ emission = 525 nm) for the detection of cytoplasmic peroxides [61,62,63].
2. Dihydrorhodamine 123 (DHR123) (5 mM, fluorescent rhodamine 123, λ excitation = 488 nm, λ emission = 525 nm) to detect levels of hydrogen peroxide in cytoplasm and mitochondria [61,62,64].
3. Mitochondria peroxy yellow 1 (MitoPY1) (1 mM, λ excitation = 488 nm, λ emission = 625 nm) to detect mitochondrial hydrogen peroxide [65].

The experimental procedure was carried out on the day of sacrifice. Whole blood was collected in EDTA tubes from adult (10-month-old) control mice and aged (22-month-old) mice, both with and without supplementation. These tubes were immediately transported to the cytometry laboratory, where the following protocol was followed.

For each marker and its respective positive and negative controls, 50 μL of blood were aliquoted into separate 12 × 75 mm cytometry tubes. Each sample was then incubated with 2 μL of Brilliant Violet 421™ anti-mouse CD45 conjugate for 15 min at room temperature in the dark. Following leukocyte labeling, firstly, erythrocytes were eliminated using the red blood cell (RBC) lysis method. eBioscience™ 10 × erythrocyte lysis buffer (Invitrogen, Thermo Fisher Scientific, Madrid, Spain) was added to the CD45-labeled blood samples, bringing the total volume to 2.5 mL. Immediately after lysis, 300 μL of lysed blood was transferred to each cytometry tube (for markers, positive controls, and negative controls), after which the specific reagents required for the experimental objectives were added.

At this stage, H_2_DCF-DA, DHR123, and MitoPY1 were added to the tubes designated for oxidative stress parameter analysis, ensuring the optimal final concentration for each fluorophore. No markers were added to the negative controls to rule out autofluorescence, while plumbagin (Pb) was added to the positive control tubes at a final concentration of 2.24 μg/mL to increase O_2_^−^ levels. Pb was preincubated with the fluorescent marker for approximately 30 min before use.

After treatment, the tubes were incubated at 37 °C for 30 min in the dark to allow proper fluorophore interaction. Technical duplicates were performed.

Finally, samples were analyzed by flow cytometry using the FACSAria III cytometer (BD Biosciences). Mononuclear cells were sorted based on their morphological characteristics, specifically size (Forward Scatter, FSC) and granularity (Side Scatter, SSC), using a linear scale derived from light scattering. A size discriminator (FS) was applied with a threshold value of approximately 25,000.

For the induced oxidative stress experiment, leukocytes (WBC) samples were subjected to oxidative stress conditions by adding inducers such as t-BHP 50 µm, which is widely used in many studies as a model compound of hydroperoxidation. During a 30 min period, blood samples were incubated with t-BHP. After that, the previous named fluorochrome were added at the same concentration and incubation time that in basal study.

### 4.6. Statistical Methods

Values are expressed as the mean ± standard error of the mean (SEM). All statistics were performed using the GraphPad Prism 8 version 9.0.0 (GraphPad software, San Diego, CA, USA).

First of all, the Shapiro–Wilk test was carried out in order to assess a normal or non-normal distribution of the samples. In case of non-normal distribution, the Kruskal–Wallis non-parametric test was used to search for statistical differences between all groups, and then the Mann–Whitney test determined significances among each pair of groups in the study. When samples showed normality, one-way ANOVA was performed to find significant differences between the groups, followed by Levene’s test for equality of variances. If the test reported distinct variances, Welch’s test was used to find significances among paired groups. If there was equality of variances, statistical differences were proved with the two-tailed Student’s *t*-test. For all data, a *p* < 0.05 was accepted as significant.

## 5. Conclusions

Our study demonstrates that PE supplementation in aged mice reduces oxidative stress in WBC, as shown by decreased basal ROS levels and enhanced resistance to induced oxidative damage. These effects were observed at both cytoplasmic and mitochondrial levels, indicating the antioxidant potential of PE in aged immune cells. This work provides new insights into the redox-modulating properties of PE and supports its potential as a nutritional strategy to promote cellular resilience during aging. Further studies are needed to investigate the underlying molecular mechanisms and to determine whether these benefits extend to other cell types or physiological systems.

## Data Availability

Data is contained within the article.

## Supplementary Materials

The following supporting information can be downloaded at: https://www.mdpi.com/article/10.3390/ijms26135957/s1.
