# Supplementary material for: Pomegranate Extract Modulates Oxidative Stress by Reducing Basal ROS Levels and Protecting White Blood Cells from Induced Oxidative Damage in Aging Mice
Source: Int J Mol Sci. 2025 Jun 20;26(13):5957. doi: 10.3390/ijms26135957 (PMC12249682; doi:10.3390/ijms26135957)
Supplement: Supplementary file 1 [file ijms-26-05957-s001.zip › ijms-3684558-supplementary.pdf]

Supplementary Table S1. Composition and analytical characterization of Pomanox® P30 extract

Component	Content (per kg)
α-Punicalagin	174.4 g
β-Punicalagin	206.8 g
α-Punicalin	5.92 g
β-Punicalin	6.48 g
Ellagic acid	7.3 g
Ellagic acid glucoside	4.2 g
Ellagic acid rhamnoside	1.3 g
Delphinidin-3,5-diglucoside	302.9 mg
Cyanidin-3,5-diglucoside	425.3 mg
Delphinidin-3-glucoside	75.7 mg
Cyanidin-3-glucoside	241.8 mg
Pelargonidin-3-glucoside	99.5 mg
**Total polyphenols (Folin–Ciocalteu)**	**61.96%**

Pomanox® P30 (Lot 0100531601) is standardized to >50% total polyphenols and punicalagins α + β ≥ 30% p/p.

The analysis of Pomanox® was performed in Euromed Quality Control laboratory. They used HPLC-UV liquid chromatography technique with a diode array UV detector (Diode Array Detector, DAD).
